# Intermittent Preventive Treatment in Pregnant Women Is Associated with Increased Risk of Severe Malaria in Their Offspring

**DOI:** 10.1371/journal.pone.0056183

**Published:** 2013-02-25

**Authors:** Whitney E. Harrington, Robert Morrison, Michal Fried, Patrick E. Duffy

**Affiliations:** 1 Seattle Children's Hospital/University of Washington School of Medicine, Department of Pediatrics, Seattle, Washington, United States of America; 2 Seattle Biomedical Research Institute, Seattle, Washington, United States of America; 3 National Institute of Allergy and Infectious Diseases, National Institutes of Health, Rockville, Maryland, United States of America; Osaka University, Japan

## Abstract

**Background:**

In areas of widespread sulfadoxine-pyrimethamine resistance, intermittent treatment in pregnancy (IPTp) fails to prevent placental malaria (PM) and may exacerbate drug resistant infections. Because PM predicts increased susceptibility to parasitemia during infancy, we hypothesized that IPTp would also increase susceptibility to malaria infection and disease in the offspring.

**Methods:**

In a birth cohort from NE Tanzania, we evaluated the association between maternal IPTp use and risk of parasitemia and severe malaria in the offspring. Using Cox Proportional Hazards Models as well as Generalized Estimating Equations, we evaluated the effects of IPTp on the entire cohort and on subgroups stratified by PM status at delivery.

**Results and Conclusions:**

Offspring of PM+ women who received IPTp had a dose-dependent decrease in time to first parasitemia (AHR = 2.13, p = 0.04 [95%CI: 1.04, 4.38]). Among all offspring, IPTp was associated with earlier first severe malaria episode (AHR = 2.32, p = 0.02 [95%CI: 1.12, 4.78]) as well as increased overall odds of severe malaria (AOR = 2.31, p = 0.03 [95%CI: 1.09, 4.88]). Cost-benefit analyses of IPTp regimens should consider the long term effects on offspring in addition to pregnancy outcomes.

## Introduction

Malaria in pregnancy is associated with numerous poor outcomes including maternal anemia [1], [2] and hypertension [3], stillbirth, premature delivery, intra-uterine growth retardation [2], low birth weight [1], [2], and perinatal death [4]. In addition to poor delivery outcomes, placental malaria (PM) is also associated with increased susceptibility to parasitemia [5], [6] and clinical malaria during infancy [5], [7] with regard to both time to first event and overall risk. Evidence suggests that this effect may be most prominent among offspring of multigravid women [6], [7]. A conclusive explanation has yet to be determined but may involve alterations in maternally transmitted malaria-specific antibodies [5] or prenatal immune priming against malaria antigens [8], [9], [10], [11].

Intermittent preventive treatment (IPTp) with sulfadoxine-pyrimethamine (SP) is used to prevent many of the harmful consequences of malaria in pregnancy, an approach that has been successful in areas of low resistance [12]. However, in an area of widespread resistance we recently observed that IPTp did not reduce the odds of PM and failed to improve other delivery outcomes [13]. In addition, among PM+ women in this population, IPTp was associated with increased drug resistance alleles, placental parasite density, and inflammation [14]. These findings are consistent with parasite competitive facilitation, a phenomenon where drug pressure eliminates drug susceptible parasites, allowing drug resistant parasites to overgrow [15].

Based on the exacerbation of PM by IPTp and the association between PM and risk of parasitemia during infancy, we hypothesized that maternal IPTp exposure might increase the risk of malaria infection and disease in their offspring. To test this hypothesis we evaluated the relationship between maternal IPTp use and risk of parasitemia and severe malaria in their offspring. We considered the possibility that the effects of IPTp might be restricted to offspring of PM+ women and therefore stratified by maternal PM status, and we additionally evaluated effect modification by parity.

## Methods

### Ethics Statement

The study was approved by both United States (Western Institutional Review Board) and Tanzanian (National Institute for Medical Research, Medical Research Coordinating Committee) ethical review boards. Written informed consent was obtained from all women for themselves and their offspring; no verbal consents were used.

### Clinical Cohort

Data and samples were collected from a previously described prospective birth cohort in Muheza, Tanzania that was conducted from 2002 to 2006 [6], [14]. Women were enrolled at delivery, and their children were followed for up to a maximum age of 192 weeks (median 112 weeks). Women and their offspring were excluded if they had chronic illness, HIV, sickle cell disease (HgbSS), or multiple gestations, resulting in consideration of 882 women and their offspring [6], [13]. The method for PM diagnosis has previously been described [14]: placental blood was obtained by manual compression of the placenta using EDTA as an anticoagulant and Giemsa-stained thick and thin blood films were prepared [16]; PM was diagnosed by microscopy of thick films. Placental inflammation was determined by histology, and the mean fractions of drug resistance alleles in individual placental infections were determined using pyrosequencing, both as previously described [14].

Determination of IPTp exposure has been previously described [13], [14]: if IPTp use was reported by any source (self-report, village health worker records, or antenatal care (ANC) card) a woman was considered to have used IPTp. In total, maternal IPTp exposure was established for 820 women and was verified by assaying for sulfa in peripheral plasma collected at delivery [13].To verify associations, we also examined the effect of increasing IPTp doses on outcomes among the subset of 371 women with ANC card documentation of the number of doses received.

For the entire cohort, there were 38,261 bloodsmears; for the 820 offspring with known maternal IPTp status there were 38,034 bloodsmears, including both routine (every two weeks for the first year of life and every four weeks thereafter) and non-routine (village-health worker visits, walk-in visits, hospitalizations) visits. A positive parasitemia was any non-zero parasite count by microscopy. Severe malaria was based on WHO criteria and included respiratory distress, severe anemia, prostration, and convulsions, but did not include hyperparasitemia that did not otherwise meet criteria for severe malaria [17], [18]. To account for possible inflation by repeat bloodsmears, any positive bloodsmears that occurred within 28 days of an initial parasitemia without an intervening negative blood smear were excluded from analysis. For the cohort of 820 offspring, this resulted in the exclusion of 1,568 bloodsmears, resulting in consideration of 36,466 visits of which 30,841 (84.6%) were routine and 5,625 (15.4%) were non-routine.

### Statistical Analysis

The primary analyses examined the association between reported IPTp and time to first malaria events and overall odds of malaria outcomes in the entire cohort, as well as stratified by maternal PM status. Any association at the p< = 0.05 level was further investigated for dose-dependent effects using ANC dose data where known. Finally, exploratory analysis considered effect modification by parity. All outcome analyses used robust standard errors to account for non-normally distributed data.

Survival analysis was conducted using Cox Proportional Hazards models to assess time to first parasitemia and time to first severe malaria. Odds of malaria outcomes (parasitemia, severe malaria) throughout early life were assessed using Generalized Estimating Equation (GEE) models with clustering by individual, a binomial outcome structure, and an exchangeable correlation matrix. Odds of parasitemia considered all visits as either a negative parasitemia (0) or positive parasitemia (1). Odds of severe malaria analysis was restricted to positive bloodsmears, considering positive bloodsmears that were not severe malaria episodes (0) and positive bloodsmears that were severe malaria episodes (1).

Covariates considered for multivariate models included sex of the child, sickle cell trait, maternal age, PM, parity, the interaction term between PM and parity [6], malaria transmission season at birth, village, bed net use, and year of enrollment into the study. Sex of child was modeled as dichotomous; sickle cell trait as categorical (HgbAA, HgbAS, unknown); maternal age as a continuous; PM as dichotomous; parity as categorical: primigravid (0 previous births), secundigravid (one previous birth), or multigravid (two or more previous births); malaria transmission season at birth as dichotomous (low from November to April versus high from May to October, based on incidence of parasitemia among 3–12 month old offspring in Muheza); village as categorical (four options); bed net usage as categorical (no bed net, untreated bed net, insecticide-treated bed net, unknown); and year of enrollment as categorical. Univariate analysis was conducted (Cox Proportional Hazards or GEE) and each covariate was tested individually in the model. Covariates were included in the final model if they changed the estimate for IPTp by more than 10% (confounding) or were significantly related to the outcome at the α< = 0.10 level (prediction), with the exception of sickle cell trait and year of enrollment which were included in all models *a priori* due to known association with the outcomes of interest.

Final covariates included in the Cox Proportional Hazards Model of time to first parasitemia included sickle cell trait, PM (predictor), parity (predictor), the interaction term between PM and parity (predictor), village (predictor), bed net use (predictor), and year of enrollment. Final covariates included in the Cox Proportional Hazards Model of time to first severe malaria included sex of the infant (predictor), sickle cell trait (predictor), village (predictor), bed net use (predictor), and year of enrollment (confounder and predictor).

In the GEE models two additional variables were tested: 1) a time varying covariate “transmission season” which represented the malaria transmission season at each visit (high or low, as defined above), and 2) a time varying covariate for age of the child in weeks, modeled as two linear variables: one for age <24 weeks and one for age > = 24 weeks, based on prior literature showing an inflection point at the age of approximately six months with regard to the risk of severe malaria during infancy [19].

Final covariates in the GEE model of overall odds of parasitemia included sickle cell trait (predictor and confounder), PM (predictor and confounder), village (predictor and confounder), bed net use (predictor and confounder), year of enrollment (predictor and confounder), transmission season (predictor), and age of the child (predictor and confounder). Final covariates in the GEE model of overall odds of severe malaria included sickle cell trait (predictor and confounder), village (predictor and confounder), bed net use (predictor), year of enrollment (predictor and confounder), and age of the child (predictor and confounder).

## Results

### Descriptive Characteristics of the Cohort

The cohort has been described in previous publications [6], [13], [14]. Of the 882 women and offspring in the cohort, 107 (12.1%) reported no IPTp exposure, while 713 (80.8%) reported IPTp exposure, and 62 (7.0%) had unknown IPTp exposure. Where doses were recorded on ANC cards, 80 (21.6%) received no doses, 156 (42.0%) received one dose, and 135 (36.4%) received two or more doses. 717 (87.4%) were PM− at delivery, while 103 (12.6%) were PM+; 232 (28.3%) were primigravid, 186 (22.7%) were secundigravid, and 402 (49.0%) were multigravid. Neither PM status nor parity differed by IPTp use. However, women who received IPTp were less likely to live in an urban setting and were more likely to be enrolled later in the study as compared to women who did not use IPTp [13], [14], emphasizing the need to control for these variables in our adjusted models.

The average duration of follow-up was 116.9 weeks among offspring of women who did not receive IPTp and 105.8 weeks among offspring of women who received IPTp. The average number of bloodsmears was 41.3 for offspring of women who did not receive IPTp of which 6.4 were non-routine, while the average number of bloodsmears was 42.2 for offspring of women who received IPTp of which 7.2 were non-routine.

### Brief Summary of Previous Results

Our previously published work was based on the same cohort of women [13], [14]. To briefly summarize our prior findings among PM+ women, IPTp was associated with an increased mean fraction of the drug resistance allele at *dhps* codon 581 (0.36 vs. 0.13 (p = 0.02)), increased parasite density (7.3% vs. 1.9% (p = 0.003)), and increased placental inflammation [14]. Drug resistance alleles were near saturation at *dhfr* codons 51, 59, and 108 and at *dhps* codons 437 and 540 and did not vary by IPTp use [14]. Among all women in the cohort, IPTp was not associated with a reduction in odds of PM, low birth weight, or maternal anemia and instead was associated with increased odds of fetal anemia [13].

### IPTp is Associated with Earlier First Parasitemia among Offspring of PM+ Women

In the cohort of 882, 715 (81.1%) offspring experienced at least one parasitemia. Of the 820 offspring for whom maternal IPTp use was known, 668 (81.5%) experienced at least one parasitemia, 78 (72.9%) among offspring of women who did not receive IPTp and 590 (82.8%) among offspring of women who received IPTp.

**Figure 1:**
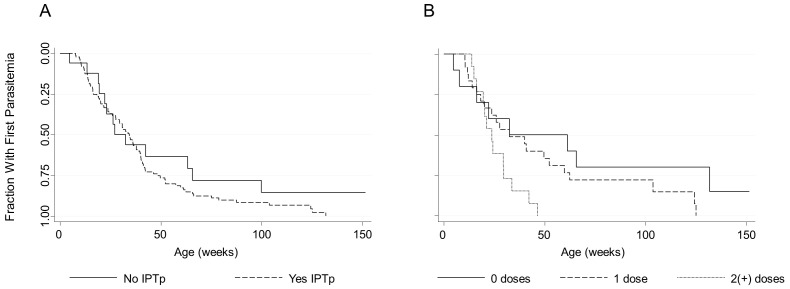
IPTp is associated with earlier first parasitemia among offspring of PM+ women. Kaplan-Meier curves of time to first parasitemia by maternal IPTp status (A) or dose (B). 1 dose: AHR = 3.71, p = 0.04 [95% CI: 1.07, 12.78]; 2(+) doses: AHR = 10.89, p = 0.003 [95%CI: 2.25, 52.78].

IPTp was not associated with time to first parasitemia in the overall cohort (AHR = 1.07, p = 0.6 [95%CI: 0.85, 1.36]) or among offspring of PM− women (AHR = 1.04, p = 0.7 [95% CI: 0.81, 1.35]). However, IPTp was associated with earlier time to first parasitemia among offspring of PM+ women (AHR = 2.13, p = 0.04 [95% CI: 1.04, 4.38]) (**Figure 1A**). This was supported by the IPTp dose data, where both one and two or more doses of IPTp were associated with earlier first parasitemia, and the hazard increased in a dose-dependent manner (test of 1 dose vs. 2(+) doses, p = 0.02) (**Figure 1B**). The association between IPTp and earlier time to first parasitemia within this group was not modified by maternal parity.

In contrast, IPTp use was not associated with overall odds of parasitemia among any group of offspring (All offspring: AOR = 1.10, p = 0.4 [95%CI: 0.90, 1.33]; offspring of PM− women: AOR = 1.13, p = 0.2 [95%CI: 0.92, 1.39]; offspring of PM+ women: AOR = 1.25, p = 0.4 [95%CI: 0.72, 2.20]).

### IPTp is Associated with Increased Risk of Severe Malaria among Offspring

Of the cohort of 882 offspring, 102 (11.6%) experienced at least one severe malaria episode. Of the 820 offspring for whom maternal IPTp use was known, 92 (11.2%) experienced at least one severe malaria episode, 8 (7.5%) among offspring of women who did not receive IPTp and 84 (11.7%) among offspring of women who received IPTp.

**Figure 2:**
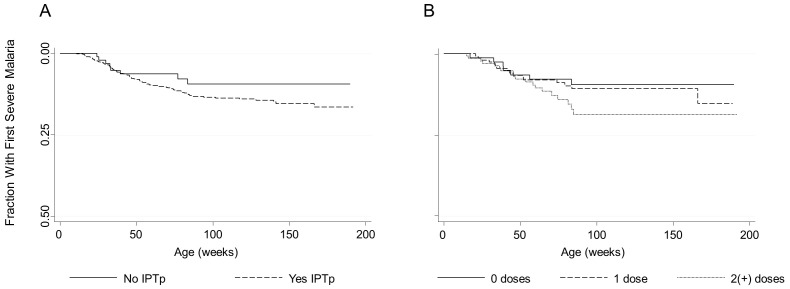
IPTp is associated with earlier first severe malaria episode. Kaplan-Meier curves of time to first severe malaria episode by maternal IPTp status (A) or dose (B). 1 dose: AHR = 1.17, p = 0.8 [95% CI: 0.45, 3.07]; 2(+) doses: AHR = 2.76, p = 0.03 [95% CI: 1.11, 6.85].

IPTp was associated with earlier first severe malaria episode among all offspring (AHR = 2.32, p = 0.02 [95%CI: 1.12, 4.78]) (**Figure 2A**). When stratified by maternal PM status this effect was non-significant within each group, likely the result of small sample sizes (offspring of PM− women: AHR = 2.08, p = 0.06 [95% CI: 0.98, 4.43]; offspring of PM+ women: AHR = 6.67, p = 0.08 [95% CI: 0.78, 56.96]). Two or more doses but not one dose was associated with earlier first severe malaria episode among all offspring (**Figure 2B**) (test of 1 dose vs. 2(+) doses, p = 0.02). The association between IPTp and earlier first severe malaria episode was not modified by maternal parity.

**Figure 3:**
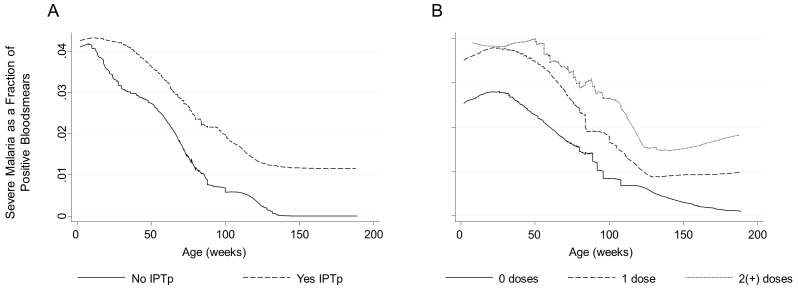
IPTp is associated with increased overall odds of severe malaria. Severe malaria as a fraction of positive bloodsmears by age of the child and maternal IPTp status (A) or dose (B), bandwidth one week. 1 dose: AOR = 1.43, p = 0.4 [95%CI: 0.62, 3.30]; 2(+) doses: AOR = 2.81, p = 0.008 [95%CI: 1.31, 6.01].

Among all offspring, IPTp was associated with increased overall odds of severe malaria (AOR = 2.31, p = 0.03 [95% CI: 1.09, 4.88]) (**Figure 3A**). When stratified by maternal PM status, the effect was no longer significant among offspring of PM− women (AOR = 1.91, p = 0.1 [95%CI: 0.88, 4.17]) but appeared to be accentuated among offspring of PM+ women (AOR = 9.32, p = 0.04 [95%CI: 1.08, 80.35]) although the model did not fully converge. The evaluation of a dose effect was limited by the small number of severe malaria cases in each stratum and the model failed to fully converge, however, two or more doses but not one dose appeared to be associated with increased odds (**Figure 3B**) (offspring of all women: test of 1 dose vs. 2(+) doses, p = 0.03). The association between IPTp and increased overall odds of severe malaria was not modified by parity.

## Discussion

We previously observed that in an area of widespread SP resistance, IPTp failed to prevent PM and instead was associated with exacerbated infections [13], [14]. We and others also observed that PM is associated with increased risk of malaria among the offspring [5], [6], [7]. We therefore hypothesized that maternal IPTp use would increase risk of malaria infection and disease among their offspring. Using a longitudinal birth cohort study, we now report that maternal IPTp use was associated with earlier first parasitemia among offspring of PM+ women in a dose-dependent fashion. IPTp was also associated with earlier first severe malaria episode and increased overall odds of severe malaria among all offspring in the cohort. This is the first report to describe an association between IPTp use in pregnant women and increased susceptibility to malaria in their offspring.

The association between IPTp and time to first parasitemia was restricted to offspring of PM+ women. This finding is consistent with a mechanism whereby IPTp may exacerbate PM infections, which are known to predict increased risk of parasitemia and clinical malaria [5], [6], [7]. Prior work has shown that PM predicts risk of malaria most strongly among offspring of multigravid women [6], [7]; however, the association between IPTp and time to parasitemia was not restricted to offspring of multigravidae, perhaps suggesting a more general mechanism beyond the exacerbation of PM. The association between IPTp and parasitemia did not extend to increased odds of parasitemia throughout early life. The association between IPTp and earlier first severe malaria as well as increased overall odds of severe malaria extended to the offspring of all women and also was not modified by maternal parity. The effect was larger among the offspring of PM+ women, which may have partially driven the association seen within the entire cohort. However, even within the offspring of PM− women alone, the association between IPTp use and increased risk of severe malaria trended toward significance. These findings may also suggest an additional mechanism beyond the exacerbation of PM, such as modulation of the level or diversity of maternal antimalarial antibodies or prenatal immune priming.

Limited evidence in humans suggests that IPTp may reduce the level of maternal antimalarial antibodies, although this may be modulated by parity [20] and HIV status [21] and is of unclear clinical significance [21], [22]. In addition, it is possible that IPTp may reduce the diversity of maternal antimalarial antibodies and as a result increase offspring susceptibility to a heterogeneous parasite population. In mice, treatment of malaria infections prior to pregnancy is associated with decreased antimalarial antibodies in dams, resulting in decreased antimalarial antibodies, increased parasitemia, and increased mortality in their malaria-infected pups [23]. In the present cohort, the difference in risk of severe malaria between offspring whose mothers did or did not receive IPTp appeared to develop around six months of age, when maternal antimalarial antibodies have significantly waned and infant immune responses take precedence [19]. The nature (tolerance or sensitization) and degree of *in utero* priming against malaria is modulated by parity and PM status [10], [24] and may be further modulated by maternal IPTp exposure. In particular, *in utero* malaria exposure and development of an immuno-tolerant response in neonates is associated with increased susceptibility to malaria during infancy [9], [10].

Our study is limited by its design. Because IPTp is the standard of care in most of Africa, ongoing randomized control trials of IPTp have not been ethically permissible. Our control group consisted of women who did not receive IPTp via their actions or the actions of the antenatal clinic where they received care. We used self-report to determine IPTp exposure in our primary analysis. We have previously shown that self-report is extremely accurate based on correlation with detectable sulfa levels at delivery [13]. We also confirmed our findings with secondary analyses of the subset of offspring whose maternal IPTp dose was documented in their antenatal records, and our findings were consistent between the two approaches. In addition, there may be differences between the exposed and unexposed populations. We have attempted to control for this possibility using adjusted models that account for a number of confounders and predictors, however, there is always the possibility of residual confounding from an unmeasured covariate. Finally, it is possible that the associations we describe might result from increased malaria exposure among the population of IPTp users and their offspring. Two factors make this unlikely: first, IPTp was national policy and should have been distributed without consideration of disease prevalence; second, we failed to find an association between IPTp and odds of parasitemia among the cohort as a whole, suggesting that baseline parasite transmission intensity was similar between the two groups.

We have previously shown that in Muheza, Tanzania, an area of widespread drug resistance, IPTp fails to prevent PM and may exacerbate drug resistant infections [13], [14]. We now describe an association between maternal IPTp exposure and increased risk of malaria outcomes in their offspring. We recognize that the cohort was small and experienced relatively few cases of severe malaria and that these results will need to be validated in larger cohorts. However, these findings emphasize that drug exposure in pregnant women has the potential to modulate long term susceptibility to disease in their offspring.

These conclusions have important implications for malaria control efforts world-wide. The burden of malaria is greatest among pregnant women and children [1], [25] and severe malaria is a leading cause of childhood mortality in endemic areas [25]. While IPTp improves pregnancy outcomes in areas of drug susceptibility, in areas of widespread resistance it may have long term harmful effects on susceptibility to parasitemia and severe malaria in the offspring. These findings call for urgent re-evaluation of ongoing IPTp policy.
